# Different lens power calculation formulas for the prediction of refractive outcome after phacoemulsification with silicone oil removal

**DOI:** 10.1186/s12886-022-02304-2

**Published:** 2022-02-13

**Authors:** Yanan Hou, Lei Liu, Gang Wang, Junwei Xie, Yi Wang

**Affiliations:** 1 Jinan University, Guangzhou, China; 2 Department of Fundus, Hefei Aier Eye Hospital, Hefei, China; 3 Department of Fundus, Chongqing Aier Eye Hospital, Chongqing, China

**Keywords:** Silicone oil tamponaded eye, Silicone oil removal, Axial, Lens assessment, intraocular lens implantation, IOL power evaluation

## Abstract

**Background:**

Formulas predicting intraocular lens power have not been compared in silicone oil-tamponaded eyes. The study aims to compare six intraocular lens power assessment formulas in silicone oil-tamponaded eyes.

**Methods:**

This prospective study included patients with silicone oil-tamponaded eyes scheduled for silicone oil removal, phacoemulsification, and intraocular lens implantation at Chongqing Aier Eye Hospital (June 2019 to December 2019). Implanted intraocular lens power was used to predict postsurgical spherical equivalence using SRK/T, Holladay 1, Holladay 2, Haigis, Hoffer Q, and Barrett Universal II, and assess those formula’s predictive accuracy with predictive error.

**Results:**

The analysis included 47 eyes in 47 patients (28 and 19 eyes with normal and long axial length, respectively). Postoperative spherical equivalence at 6 months in normal and long axial length eyes was − 0.6 ± 0.96 and − 0.8 ± 1.52 D, respectively. Predictive error values for SRK/T, Holladay 1, Holladay 2, Haigis, and Hoffer Q and Barrett Universal II were − 0.18 ± 0.92, − 0.15 ± 0.88, − 0.06 ± 0.94, − 0.15 ± 0.87, and − 0.05 ± 0.90 D and − 0.06 ± 0.90, respectively, for normal axial length eyes and 0.15 ± 1.16, 0.46 ± 1.17, 0.28 ± 1.11, − 0.04 ± 1.12, 0.49 ± 1.09 D and 0.11 ± 0.99, respectively, for long axial length eyes. For normal axial length eyes, predicted outcomes were similar to actual outcomes for all formulas. For long axial length eyes, predicted outcomes differed significantly from measured postsurgical values for Holladay 1, Holladay 2, and Hoffer Q (*P* < 0.05) but not SRK/T or Haigis or Barrett Universal II .

**Conclusions:**

The formulas had comparable predictive accuracy in silicone oil-tamponaded eyes with normal axial length, but Haigis or SRK/T or Barrett Universal II may be preferable in long axial length eyes.

**Trial registration:**

ChiCTR1900023215.

## Background

Silicone oil is widely used in the clinic to treat complex retinal detachment associated with proliferative vitreoretinopathy, giant retinal tears, and ocular trauma [1–3]. However, the long-term use of silicone oil tamponade (SOT) is associated with a reduction in the opacity of the lens nucleus [4] and a high rate of cataract formation after 6–12 months, which causes a deterioration in vision and impairs visualization of the fundus [5, 6]. Therefore, silicone oil removal (SOR), cataract surgery, and intraocular lens (IOL) implantation are usually performed as a single procedure 3–6 months after SOT [7–9].

Cataract surgery with IOL implantation requires the accurate determination of IOL power to minimize postsurgical refractive errors [10, 11]. More than 20 different formulas for calculating IOL power are currently available, including Barrett Universal II, Haigis, Holladay 1, Holladay 2, SRK/T, Hoffer Q, and T2. Despite equivalence in some settings, the relative performances of these various formulas can vary depending on the clinical features of the eye undergoing surgery and the type of lens being implanted. For example, Barret Universal II has been reported to be superior to Holladay 2, SRK/T, Hoffer Q, and Holladay 1 in the prediction of IOL power in long eyes [12, 13]. Furthermore, Haigis may have advantages over Hoffer Q, SRK/T, and SRK II for predicting IOL power in short eyes [14], although equivalence between Barrett Universal II, Haigis, Hill-RBF, Hoffer Q, Holladay 1, Holladay 2, and Olsen in short eyes have also been reported [15]. Additionally, SRK/T was found to be more accurate than Barrett Universal II, Haigis, Hoffer Q, and Holladay 1 for eyes with keratoconus [16], while Barrett Universal II, Hill-RBF, Olsen, and T2 have been recommended over Hoffer Q, Holladay 1, Holladay 2 and SRK/T for quadrifocal intraocular lenses [17].

Unfortunately, silicone oil alters the refraction of the eye because it has a high refractive index [18] and can change the structure of the eye. This makes it difficult to accurately calculate IOL power in eyes with SOT, which increases the risk of residual refractive errors after surgery. Prior research has shown that it is more challenging to predict the potential effective lens position and refractive outcomes in eyes subjected to vitrectomy and SOT [19, 20]. Previous studies evaluating cataract surgery in combination with silicone oil removal have focused mainly on the postoperative refractive status and precise measurement of axial length [21–23]. However, to the best of our knowledge, no studies have reported data comparing formulas for predicting IOL power in eyes with SOT. The availability of such data would help clinicians to select the optimal formula for use in patients with SOT undergoing SOR, cataract surgery, and IOL implantation.

Therefore, the aim of this study was to use the IOLMaster 700 (Carl Zeiss, Oberkochen, Germany) to compare the accuracy of five IOL power assessment formulas (SRK/T, Hoffer Q, Haigis, Holladay 1 and Holladay 2 and Barrett Universal II) in the prediction of IOL power in eyes filled with silicone oil.

## Methods

### Study design and participants

This prospective, single-center case series included patients who had previously undergone SOT for retinal detachment and were scheduled to undergo SOR and phacoemulsification surgery for cataracts between June 2019 and December 2019 at Chongqing Aier Eye Hospital. The study was performed in accordance with the principles of the Declaration of Helsinki, and signed informed consent was provided by each participant in accordance with the Ethics Committee of Chongqing Aier Eye Hospital (EC approval number: IRB2019008). This trial is registered at the Chinese Clinical Trial Registry (ChiCTR1900023215).

The inclusion criteria were 1) intravitreal silicone oil in one eye for previous retinal detachment (confirmed by a history of ophthalmic surgery, slit-lamp biomicroscopy and indirect ophthalmoscopic examination), 2) normal macular morphology for the attached retina, 3) cataract, 4) corrected distance visual acuity (CDVA) > 0.1 in the eye with SOT based on assessment with an international standard visual acuity chart, and 5) clear media in both eyes. The exclusion criteria were 1) keratopathy or diabetic retinopathy, 2) macular edema or hole, 3) glaucoma, 4) secondary epiretinal membrane, 5) retinal re-detachment during observation, 6) previous refractive surgery, 7) axial length not reliably assessable by partial coherence interferometry, or 8) pre- and intraoperative complications preventing IOL placement in the capsular bag.

The design of the International visual acuity chart followed the Weber-Fechner rule. There were 14 lines spaced by 24 mm. The optotype was based on the equal length of the three lines of E, with an increment rate of $\sqrt{10}$. The test was performed at a distance of 5 m. The VA was recorded using the 5-grade recording method and the decimal recording method. The 5-grade recording method could be directly used for VA statistics and is equivalent to the logMAR recording method.

### Preoperative assessments

The axial length was measured by partial coherence interferometry (IOLMaster 700 using the SO-filled eyes program; Carl Zeiss) with the patient in the upright position [24]. Predicted spherical equivalent (SE) values were determined preoperatively using the SRK/T [25], Holladay 1 [26], Holladay 2 [27], Haigis [28], and Hoffer Q [29] and Barrett Universal II formulas.

### Sor

SOR under retrobulbar anesthesia was performed by experienced retinal surgeons (L.L. and G.W., Deputy Chief Physicians with more than 10 years of relevant surgical experience) using a Constellation device (Alcon, Inc., Fort Worth, TX, USA). SOR was carried out via the pars plana.

### Phacoemulsification and IOL implantation

Phacoemulsification was performed first, the intraocular lens was implanted, and then SOR was performed. A 2.75-mm micro-coaxial corneal incision was made, and capsulorhexis was performed. The lens was emulsified and then replaced with an IOL. Two types of IOL were used: SN60WF (Alcon, Inc.) and AR40E (AMO, Inc., Santa Anna, CA, USA). The IOL was inserted into the capsular bag without a capsular tension ring in all cases. Postoperative treatment consisted of routine topical antibiotics and anti-inflammatory agents for 1 month.

### Follow-up and assessments

Follow-up was carried out up to 6 months postoperatively. CDVA and intraocular pressure (IOP) were measured daily during hospitalization; the CDVA assessment was made using, International standard visual acuity chart, and the data were transformed into logarithm of the minimum angle of resolution (logMAR) units. The following clinical data were obtained from the medical records: age, sex, axial length, time from SOT to phacoemulsification, target refraction (predicted refraction), and postsurgical SE at 6 months (actual refraction). The prediction error (PE) for each formula was calculated as PE = actual refraction after surgery - predicted refraction. The percentages of cases with absolute PE values within 0.25 diopter (D), 0.5 D, 0.75 D, and 1.0 D were determined.

### Statistics analysis

Data analysis was performed using SPSS 23.0 (IBM Corp., Armonk, NY, USA). Descriptive statistics (e.g., mean ± standard deviation) were used to evaluate the predictive accuracies of the various formulas in comparison with postsurgical data (PE values). The nonparametric Wilcoxon signed-rank test was carried out to compare the predicted refraction values with postsurgical measurements. One-way analysis of variance (ANOVA) was performed to compare PEs among the different formulas. In addition, the chi-squared test was used to compare the percentage of cases with PEs within ±0.25 D, ±0.5 D, ±0.75 D, and ± 1.00 D of the postsurgical refraction between the various formulas. *P* < 0.05 was considered statistically significant.

## Results

### Patient characteristics

Of the 78 patients initially screened for inclusion, 31 patients were excluded due to loss to follow-up (*n* = 18), macular edema (*n* = 5), secondary epiretinal membrane (*n* = 3), intraocular lens displacement (*n* = 2), macular hole (*n* = 1), vitreous hemorrhage (*n* = 1) and retinal re-detachment (*n* = 1). Normal axial Length was defined as an axial length from 22 to 26 mm [30]. A long axial length was defined as > 26 mm. Therefore, 47 eyes in 47 patients (28 eyes with a normal axial length and 19 eyes with a long axial length) successfully treated with a combination of SOR, phacoemulsification, and IOL implantation were included in the analysis. The type of IOL implanted was SN60WF in 9 eyes (19.1%) and AR40E in 38 eyes (80.9%). The participants’ demographic features and preoperative data are reported in Table 1. The time from SOT to phacoemulsification was 3.81 ± 1.32 months for eyes with normal axial length and 4.32 ± 1.27 months for eyes with a long axial length. The axial lengths of the eyes with normal and long axial lengths were 24.25 ± 0.96 mm and 28.50 ± 1.66 mm, respectively.

**Table 1:** Demographic and preoperative characteristics of patients with silicone oil tamponade

Characteristic	Normal axial length	Long axial length
**Eyes, n**	28	19
**Age, years**	60 ± 8.8	51.8 ± 5.0
**Sex (male/female), n**	15/13	11/8
**Axial length (mm)**	24.25 ± 0.96	28.50 ± 1.66
**CDVA, logMAR**	0.80 ± 0.31	0.84 ± 0.33
**IOP before SOR (mmHg)**	15.32 ± 3.60	19.32 ± 2.70
**Duration of SOT (months)**	3.81 ± 1.32	4.32 ± 1.27

Data are presented as the mean ± standard deviation unless otherwise stated. *CDVA* Corrected distance visual acuity, *IOP* Intraocular pressure, *logMAR* Logarithm of the minimum angle of resolution, *SOR* Silicone oil removal, *SOT* Silicone oil tamponade

### Visual acuity

The CDVA value before cataract/SOR surgery was 0.80 ± 0.31 logMAR in eyes with normal axial length and 0.84 ± 0.33 logMAR in eyes with long axial length. CDVA was significantly decreased after surgery to 0.55 ± 0.34 logMAR in eyes with normal axial length (*P* < 0.05) and to 0.60 ± 0.39 logMAR (*P* < 0.05) in eyes with long axial length.

### Predictive accuracies of the various IOL power assessment formulas

Tables 2 and 3 summarize data for the predictive accuracies of the individual IOL power assessment formulas for normal axial length eyes and long axial length eyes, respectively. Postsurgical SE values were − 0.60 ± 0.96 D for eyes with normal axial length and − 0.80 ± 1.52 D for eyes with long axial length. PE values for the SRK/T, Holladay 1, Holladay 2, Haigis, Hoffer Q and Barrett Universal II formulas were − 0.18 ± 0.92 D, − 0.15 ± 0.88 D, − 0.06 ± 0.94 D, − 0.15 ± 0.87 D, − 0.05 ± 0.90 D and − 0.06 ± 0.90D, respectively for normal axial length eyes and 0.15 ± 1.16 D, 0.46 ± 1.17 D, 0.28 ± 1.11 D, − 0.04 ± 1.12 D, 0.49 ± 1.09 D and 0.11 ± 0.99, respectively, for long axial length eyes. The refractive outcomes predicted by the various formulas for eyes with normal axial length were similar to the actual postoperative SE values (all *P* > 0.05). However, for eyes with long axial length, Holladay 1, Holladay 2, and Hoffer Q yielded predicted refractive outcomes that differed significantly from the measured postsurgical SE values (*P* < 0.05), in contrast to SRK/T (*P* = 0.121) Haigis (*P* = 0.355) and Barrett Universal II (*P* = 0.268). In addition, the mean and absolute PEs were similar for all formulas (all *P* > 0.05).

**Table 2:** Predictive accuracies of the various IOL power assessment formulas for eyes with normal axial length

Formula	Predicted SE, D	Mean prediction error, D^**#**^	***P***-value†	Absolute error, D
**SRK/T**	−0.40 ± 0.38	−0.18 ± 0.92	0.16	0.74 ± 0.53
**Holladay 1**	− 0.46 ± 0.42	−0.15 ± 0.88	0.17	0.73 ± 0.49
**Holladay 2**	−0.55 ± 0.46	−0.06 ± 0.94	0.39	0.73 ± 0.56
**Haigis**	− 0.45 ± 0.48	−0.15 ± 0.87	0.19	0.72 ± 0.49
**Hoffer Q**	− 0.54 ± 0.51	−0.05 ± 0.90	0.39	0.71 ± 0.54
**Barrett Universal II**	− 0.52 ± 0.46	− 0.06 ± 0.90	0.32	0.72 ± 0.52
*P*-value*		0.983		0.999

Values are presented as the mean ± standard deviation. IOL, intraocular lens; SE, spherical equivalent; ^#^Postoperative SE - predicted SE. †*P*-values derived by the Wilcoxon signed-rank test; *P-values* obtained by one-way analysis of variance

**Table 3:** Predictive accuracies of the various IOL power assessment formulas for eyes with long axial length

Formula	Predicted SE, D	Mean prediction error, D^**#**^	***P***-value†	Absolute error, D
**SRK/T**	− 0.95 ± 0.63	0.15 ± 1.16	0.121	0.78 ± 0.67
**Holladay 1**	−1.26 ± 0.63	0.46 ± 1.17	0.015	0.94 ± 0.63
**Holladay 2**	− 1.08 ± 0.65	0.28 ± 1.11	0.04	0.80 ± 0.62
**Haigis**	−0.77 ± 0.70	−0.04 ± 1.12	0.355	0.63 ± 0.73
**Hoffer Q**	−1.28 ± 0.77	0.49 ± 1.09	0.009	0.88 ± 0.63
**Barrett Universal II**	−1.03 ± 0.68	0.11 ± 0.99	0.268	0.77 ± 0.68
*P*-value*		0.492		0.594

Values are presented as the mean ± standard deviation. IOL, intraocular lens; SE, spherical equivalent; ^#^Postoperative SE - predicted SE. †*P*-values derived by the Wilcoxon signed-rank test; *P-values* obtained by one-way analysis of variance

### Percentages of cases with absolute PE values within ±0.25 D, ±0.50 D, ±0.75 D, and ± 1.00 D

Table 4 (normal axial length eyes) and Table 5 (long axial length eyes) show the percentages of cases with absolute PE values within various diopter ranges for each formula. For both normal axial length eyes and long axial length eyes, Hoffer Q and Haigis appeared to yield the highest percentages of eyes with a predicted refractive error within ±0.50 D of the measured postsurgical refraction. However, statistical comparisons using the chi-squared test did not demonstrate any significant differences between formulas (see Tables 4 and 5).

**Table 4:** Percentages of normal axial length eyes within various diopter ranges of the outcomes predicted

Range (D)	SRK/Tn (%)	Holladay 1n (%)	Holladay 2n (%)	Haigisn (%)	Hoffer Qn (%)	***Barrett Universal II***	***P***
±0.25	3 (10.7)	4 (14.3)	5 (17.9)	4 (14.3)	6 (21.4)	4 (14.3)	0.842
±0.50	11 (39.3)	12 (42.9)	11 (39.3)	8 (28.6)	13 (46.4)	11 (39.3)	0.707
±0.75	18 (64.3)	18 (64.3)	18 (44.3)	19 (67.9)	16 (57.1)	19 (67.9)	0.942
±1.00	21 (75.0)	23 (82.1)	22 (78.6)	23 (82.1)	23 (82.1)	22 (78.6)	0.941
> 1.00	7 (25.0)	5 (17.9)	6 (21.4)	5 (17.9)	5 (17.9)	6 (21.4	0.952

**Table 5:** Percentages of long axial length eyes within various diopter ranges of the outcomes predicted

Range (D)	SRK/Tn (%)	Holladay 1n (%)	Holladay 2n (%)	Haigisn (%)	Hoffer Qn (%)	***Barrett Universal II***	***P***
±0.25	3 (15.8)	1 (5.3)	3 (15.8)	6 (31.6)	2 (10.5)	3 (15.8)	0.235
±0.50	5 (26.3)	6 (31.6)	8 (42.1)	10 (52.6)	7 (36.8)	10 (52.6)	0.512
±0.75	12 (63.2)	8 (42.1)	10 (52.6)	14 (73.7)	9 (47.4)	12 (63.2)	0.287
±1.00	14 (73.7)	11 (57.9)	13 (68.4)	16 (84.2)	12 (63.2)	14 (73.7)	0.461
> 1.00	5 (26.3)	8 (42.1)	6 (31.6)	3 (15.8)	7 (36.8)	5 (26.3)	0.455

## Discussion

To the best of our knowledge, the present prospective study is the first to directly compare commonly used formulas for predicting postoperative SE in eyes with SOT. A notable finding of our study was that the outcomes predicted by all five (six) formulas were similar to the actual outcomes in normal axial length eyes. However, in long axial length eyes, the predicted outcomes differed significantly from the actual postsurgical values for Holladay 1, Holladay 2, and Hoffer Q, whereas no significant difference was observed for SRK/T and Haigis and Barrett Universal II. Taken together, our research provides preliminary evidence that although the six formulas had comparable predictive accuracy in eyes with normal axial length and SOT, Haigis or SRK/T or Barrett Universal II may be preferable to Holladay 1, Holladay 2, and Hoffer Q in long axial length eyes with SOT.

This study used a swept-source optical biometer (IOLMaster-700, Chinese version, Carl Zeiss, Oberkochen, Germany) [31] to assess the ability of various IOL calculation formulas to predict postoperative SE in normal and long axial length eyes filled with silicone oil. The accuracy of IOL power calculations is mostly dependent on three parameters: presurgical biometric information (axial length, anterior chamber depth [ACD], lens thickness, and keratometric index [K]), the IOL power assessment formula used, and manufacturer-reported IOL power quality control [32]. As far as preoperative biometric data are concerned, previous studies have demonstrated that the IOLMaster provides high accuracy and limited deviation in the prediction of postsurgical refractive errors in eyes with SOT [33, 34]. The IOLMaster measures all biometric indexes, and distinct formulas for IOL power assessment are available. The optical biometry system determines ACD, K, and axial length using dual-beam partial coherence interferometry [35] to assess the infrared laser’s reflection from internal tissue interfaces, i.e., the optical path length between the cornea’s anterior surface and the retinal pigment epithelium.

Besides precise biometry, the accurate prediction of postsurgical refractive outcomes relies on the use of an appropriate IOL power assessment formula. Studies assessing the efficacies of different formulas in silicone oil-filled eyes are scarce, indicating the need for further research to evaluate the accuracies of various formulas in this clinical setting. In third-generation formulas, including Holladay 1, SRK/T, and Hoffer Q, postsurgical ACD depends on axial length and corneal curvature [25, 26, 29]. Numerous studies have shown that the fourth-generation IOL power assessment formulas (Holladay 2, Barrett Universal II, Olsen, and Haigis) have high accuracy in adult patients [12–17, 36]. However, there is a paucity of data comparing the performances of different formulas in eyes with SOT. In addition, previous studies were performed in eyes without SOT, impairing comparisons with the present study.

The present study evaluated three third-generation formulas (Holladay 1, SRK/T, and Hoffer Q) and three fourth-generation formulas (Haigis and Holladay 2 and Barrett Universal II) preinstalled on the IOLMaster 700 in patients with SOT eyes. Furthermore, recent protocols comparing the accuracy of these formulas were applied [37, 38]. An important finding of our research was that the six IOL calculation formulas had comparable predictive accuracy in eyes with a normal axial length. These results are consistent with those reported previously by Narvaez et al. (comparing Holladay 2, Hoffer Q, Holladay 1 and SRK/T) [39] and Olsen et al. (comparing Haigis, Hoffer Q, Holladay 1 and SRK/T) [40] in normal axial length eyes without SOT. Our study findings suggest that any of the six formulas preinstalled on the IOLMaster 700 could be used for normal axial length eyes filled with silicone oil.

However, we also found that predicted and actual postsurgical outcomes differed significantly in long axial length eyes for Holladay 1, Holladay 2, and Hoffer Q. By contrast, Haigis and SRK/T and Barrett Universal II predicted refractive outcomes that did not differ significantly from the measured postsurgical SE in long axial length eyes. In addition, the predicted outcome in long axial length eyes was within ±0.50 D of the actual outcome in 52.6% of cases for Haigis and Barrett Universal II, but only 26.3% of cases for SRK/T. Based on these preliminary data, we tentatively recommend that the Haigis and Barrett Universal II formula should be used in long axial length eyes with SOT. In agreement with our findings, a previous study also concluded that Haigis was the best formula for predicting refractive outcomes in patients with long axial length eyes undergoing phacoemulsification and IOL implantation, although only some of the patients included in the analysis had silicone-filled eyes [41]. However, since there were no marked differences between SRK/T, Haigis, and Barrett Universal II in the prediction of residual refractive errors in long axial length eyes, we would hesitate to make a strong recommendation between these three formulas. Previous research assessing the use of SRK/T, Haigis, and Barrett Universal II in simple cataract surgery also demonstrated that these formulas were accurate and exhibited advantages in long axial length eyes [42–45]. Furthermore, the present results are partly consistent with a prior study that recommended using SRK/T or Holladay in long axial length eyes filled with silicone oil [46]. Unfortunately, this previous investigation did not include the Haigis formula in the analysis, and its conclusions regarding the Holladay formula appear not to be entirely consistent with the present findings. Possible reasons for the discrepancies between studies with regard to the Holladay formula results include differences in the sample sizes (*n* = 6 versus *n* = 19) or devices employed (perioperative A-scan biometry versus IOLMaster biometry), although another study found that A-scan immersion biometry and IOLMaster biometry had comparable accuracies and reliabilities in silicone oil-filled eyes [19]. In addition, other studies have also reported that SRK/T, Barrett Universal II, and Haigis perform better than Holladay 1 and Holladay 2 in long axial length eyes [40, 43, 47]. Therefore, further research is needed to definitively establish whether Haigis, SRK/T, or Barrett Universal II may have advantages over other formulas in the prediction of IOL power in eyes with long axial length.

This study has some limitations. First, in addition to the small sample size, only SN60WF and AR40E were used as IOLs, so the current results may not be extrapolatable to other IOL types. Second, we only analyzed the six formulas provided with the IOLMaster-700, whereas more than 20 formulas are available. Therefore, other formulas will need to be investigated in future research. Third, the sample size of short eyes filled with silicone oil was relatively small (*n* = 3 patients) and could not be included. Therefore, this study did not include a subgroup of eyes with an axial length shorter than 22 mm. Additional studies are needed to compare different formulas for short axial length eyes with SOT.

In conclusion, the Haigis, Hoffer Q, Holladay 1, Holladay 2, and SRK/T formulas were comparable in normal axial length eyes filled with silicone oil. Despite comparable absolute PE values obtained by these five formulas, SRK/T and Haigis may have advantages over the other formulas in long axial length eyes containing silicone oil.

## Data Availability

The datasets used and analyzed in the current study are available from the corresponding author on demand.

## Abbreviations

SOT: Silicone oil tamponade
SOR: Silicone oil removal
IOL: Intraocular lens
CDVA: Corrected distance visual acuity
SE: Spherical equivalent
IOP: Intraocular pressure
PE: Prediction error
ACD: anterior chamber depth

## References

[1] Szurman P, Roters S, Grisanti S, Aisenbrey S, Rohrbach JM, Warga M (2007). Primary silicone oil tamponade in the management of severe intraocular foreign body injuries: an 8-year follow-up. Retina (Philadelphia, Pa)..

[2] Schwartz SG, Flynn HW, Wang X, Kuriyan AE, Abariga SA, Lee WH (2020). Tamponade in surgery for retinal detachment associated with proliferative vitreoretinopathy. Cochrane Database Syst Rev.

[3] Adelman RA, Parnes AJ, Sipperley JO, Ducournau D (2013). Strategy for the management of complex retinal detachments: the European vitreo-retinal society retinal detachment study report 2. Ophthalmology..

[4] Koch F, Kloss KM, Hockwin O, Spitznas M (1991). Lens changes following intraocular tamponade in vitrectomy. Linear densitometric image analysis of Scheimpflug photographs 6 months after operation. Klinische Monatsblatter fur Augenheilkunde.

[5] Grewing R, Mester U (1992). Therapeutic possibilities in lens opacity after silicon oil tamponade. Klinische Monatsblatter fur Augenheilkunde..

[6] Effert R, Lommatzsch A, Wessing A (1996). Clinical experiences after implantation of various lens types in silicon oil tamponade. Klinische Monatsblatter fur Augenheilkunde..

[7] Antoun J, Azar G, Jabbour E, Kourie HR, Slim E, Schakal A (2016). Vitreoretinal surgery with silicone oil tamponade in primary uncomplicated rhegmatogenous retinal detachment: clinical outcomes and complications. Retina (Philadelphia, Pa)..

[8] Jančo L, Tkáčová Villemová K, Ondrejková M, Vida R, Bartoš M, Mesárošová M (2014). Retinal tamponade with silicone oil - long term results. Ceska Slovenska Oftalmol.

[9] Al-Habboubi HF, Al-Zamil W, Al-Habboubi AA, Khandekar R (2018). Visual outcomes and refractive status after combined silicone oil removal/cataract surgery with intraocular Lens implantation. J Ophthalmic Vis Res.

[10] Murray DC, Durrani OM, Good P, Benson MT, Kirkby GR (2002). Biometry of the silicone oil-filled eye: II. Eye (London, England)..

[11] Olsen T (2007). Calculation of intraocular lens power: a review. Acta Ophthalmol Scand.

[12] Wang Q, Jiang W, Lin T, Zhu Y, Chen C, Lin H (2018). Accuracy of intraocular lens power calculation formulas in long eyes: a systematic review and meta-analysis. Clin Exp Ophthalmol.

[13] Kane JX, Van Heerden A, Atik A, Petsoglou C (2016). Intraocular lens power formula accuracy: comparison of 7 formulas. J Cataract Refract Surg.

[14] Wang Q, Jiang W, Lin T, Wu X, Lin H, Chen W (2018). Meta-analysis of accuracy of intraocular lens power calculation formulas in short eyes. Clin Exp Ophthalmol.

[15] Gökce SE, Zeiter JH, Weikert MP, Koch DD, Hill W, Wang L (2017). Intraocular lens power calculations in short eyes using 7 formulas. J Cataract Refract Surg.

[16] Savini G, Abbate R, Hoffer KJ, Mularoni A, Imburgia A, Avoni L (2019). Intraocular lens power calculation in eyes with keratoconus. J Cataract Refract Surg.

[17] Shajari M, Kolb CM, Petermann K, Böhm M, Herzog M, de’Lorenzo N (2018). Comparison of 9 modern intraocular lens power calculation formulas for a quadrifocal intraocular lens. J Cataract Refract Surg.

[18] Dick B, Pavlovic S, Jacobi FK, Eisenmann D, Jacobi KW (1996). Objective determination of refractive changes in silicon oil filled eyes--effect of head position on refraction. Klinische Monatsblatter fur Augenheilkunde..

[19] El-Baha SM, Hemeida TS (2009). Comparison of refractive outcome using intraoperative biometry and partial coherence interferometry in silicone oil-filled eyes. Retina (Philadelphia, Pa).

[20] Falkner-Radler CI, Benesch T, Binder S (2008). Accuracy of preoperative biometry in vitrectomy combined with cataract surgery for patients with epiretinal membranes and macular holes: results of a prospective controlled clinical trial. J Cataract Refract Surg.

[21] Kas’yanov AA, Sdobnikova SV, Troitskaya NA, Ryzhkova EG, Kas’yanov AA, Sdobnikova SV (2015). Intraocular lens power calculation in silicone-filled eyes. Vestn oftalmol.

[22] Kanclerz P, Grzybowski A (2019). Accuracy of intraocular Lens power calculation in eyes filled with silicone oil. Semin Ophthalmol.

[23] Piasecka K, Bednarski M, Michalewski J, Nawrocki J, Michalewska Z (2016). Optical low-coherence reflectometry in the calculation of intraocular lens power in silicone oil-filled eyes. Klin Ocz.

[24] Greenberg PB, Havnaer A, Oetting TA, Garcia-Ferrer FJ (2012). Cataract surgery practice patterns in the United States veterans health administration. J Cataract Refract Surg.

[25] Retzlaff JA, Sanders DR, Kraff MC (1990). Development of the SRK/T intraocular lens implant power calculation formula. J Cataract Refract Surg.

[26] Holladay JT, Prager TC, Chandler TY, Musgrove KH, Lewis JW, Ruiz RS (1988). A three-part system for refining intraocular lens power calculations. J Cataract Refract Surg.

[27] Iba T, Levy JH, Levi M, Connors JM, Thachil J (2020). Coagulopathy of coronavirus disease 2019. Crit Care Med.

[28] Haigis W, Lege B, Miller N, Schneider B (2000). Comparison of immersion ultrasound biometry and partial coherence interferometry for intraocular lens calculation according to Haigis. Graefes Arch Clin Exp Ophthalmol.

[29] Hoffer KJ (1993). The Hoffer Q formula: a comparison of theoretic and regression formulas. J Cataract Refract Surg.

[30] Kim SY, Lee SH, Kim NR, Chin HS, Jung JW (2020). Accuracy of intraocular lens power calculation formulas using a swept-source optical biometer. PLoS One.

[31] Olsen T, Thorwest M (2005). Calibration of axial length measurements with the Zeiss IOLMaster. J Cataract Refract Surg.

[32] Drexler W, Findl O, Menapace R, Rainer G, Vass C, Hitzenberger CK (1998). Partial coherence interferometry: a novel approach to biometry in cataract surgery. Am J Ophthalmol.

[33] Kunavisarut P, Poopattanakul P, Intarated C, Pathanapitoon K (2012). Accuracy and reliability of IOL master and A-scan immersion biometry in silicone oil-filled eyes. Eye (London, England).

[34] Lege BA, Haigis W (2004). Laser interference biometry versus ultrasound biometry in certain clinical conditions. Graefes Arch Clin Exp Ophthalmol.

[35] Findl O, Drexler W, Menapace R, Heinzl H, Hitzenberger CK, Fercher AF (2001). Improved prediction of intraocular lens power using partial coherence interferometry. J Cataract Refract Surg.

[36] Melles RB, Holladay JT, Chang WJ (2018). Accuracy of intraocular Lens calculation formulas. Ophthalmology..

[37] Hoffer KJ, Aramberri J, Haigis W, Olsen T, Savini G, Shammas HJ (2015). Protocols for studies of intraocular lens formula accuracy. Am J Ophthalmol.

[38] Wang L, Koch DD, Hill W, Abulafia A (2017). Pursuing perfection in intraocular lens calculations: III. Criteria for analyzing outcomes. J Cataract Refract Surg.

[39] Narváez J, Zimmerman G, Stulting RD, Chang DH (2006). Accuracy of intraocular lens power prediction using the Hoffer Q, Holladay 1, Holladay 2, and SRK/T formulas. J Cataract Refract Surg.

[40] Olsen T, Hoffmann P (2014). C constant: new concept for ray tracing-assisted intraocular lens power calculation. J Cataract Refract Surg.

[41] Wang JK, Hu CY, Chang SW (2008). Intraocular lens power calculation using the IOLMaster and various formulas in eyes with long axial length. J Cataract Refract Surg.

[42] Aristodemou P, Knox Cartwright NE, Sparrow JM, Johnston RL (2011). Formula choice: Hoffer Q, Holladay 1, or SRK/T and refractive outcomes in 8108 eyes after cataract surgery with biometry by partial coherence interferometry. J Cataract Refract Surg.

[43] Bang S, Edell E, Yu Q, Pratzer K, Stark W (2011). Accuracy of intraocular lens calculations using the IOLMaster in eyes with long axial length and a comparison of various formulas. Ophthalmology..

[44] Chen C, Xu X, Miao Y, Zheng G, Sun Y, Xu X (2015). Accuracy of intraocular Lens power formulas involving 148 eyes with long axial lengths: A retrospective chart-review study. J Ophthalmol.

[45] Chong EW, Mehta JS (2016). High myopia and cataract surgery. Curr Opin Ophthalmol.

[46] el-Baha SM, el-Samadoni A, Idris HF, Rashad KM. (2003). Intraoperative biometry for intraocular lens (IOL) power calculation at silicone oil removal. Eur J Ophthalmol.

[47] Ji J, Liu Y, Zhang J, Wu X, Shao W, Ma B, et al. Comparison of six methods for the intraocular lens power calculation in high myopic eyes. Eur J Ophthalmol. 2021;31(1):96–102. 10.1177/1120672119889016 31744328

